# Letter regarding “Prospective randomized trial comparing relapse rates in dogs with steroid‐responsive meningitis‐arteritis treated with a 6‐week or 6‐month prednisolone protocol”

**DOI:** 10.1111/jvim.17188

**Published:** 2024-09-12

**Authors:** Andrew Woodward

**Affiliations:** ^1^ Independent Canberra Australian Capital Territory Australia

Dear Editors,

I read with interest the article “Prospective randomized trial comparing relapse rates in dogs with steroid‐responsive meningitis‐arteritis treated with a 6‐week or 6‐month prednisolone protocol.”^1^ I am concerned that the article contains substantial misinterpretations of statistical evidence, which undermine the reliability of the authors' conclusions.

The final paragraph of the paper contains this statement^1^:

In conclusion, given there was no significant difference between the “short” 6‐week or “long” 6‐month treatment protocols in the incidence risk of at least 1 relapse event or incidence rate of total relapse events in our study, both a “long” 6‐month or “short” 6‐week prednisolone protocol could be considered to treat SRMA cases.

The phrase “given there was no significant difference” suggests that the authors have reached their conclusion primarily based on the fact that some *P*‐value was large; specifically, that positive evidence was obtained that “long” or “short” protocols were not different, and therefore can be considered exchangeable (“both… could be considered to treat SRMA cases”).

Unfortunately, the phrase “no significant difference” may be deeply misleading unless it is correctly interpreted, under the unintuitive logic of frequentist hypothesis tests, and has led the authors to a mistake; it may be true that the interventions are practically exchangeable, but that conclusion cannot be reached from “not significant.” The *P*‐value resulting from frequentist hypothesis tests represents an approximation of the probability that data more extreme than the data at hand would be observed, if the test hypothesis was true; where “probability” represents the frequency in a hypothetical series of identical repeated trials, and the test hypothesis is some statistical model including its parameters.^2^

The *P*‐value is calculated under the assumption that the test hypothesis (whatever it is) is true, so never indicates support for the test hypothesis, which would involve circular reasoning. Though the “evidential” meaning of *P*‐values is contested and generally dubious,^3^ in simple terms the *P*‐value can be considered a summary of the evidence provided by the data to *refute* the test hypothesis,^4^ or equivalently, an expression of how surprising it would be to observe data at least as extreme as these, if the test hypothesis was in fact true.^2^ Though a small *P*‐value may suggest (charitably) that some aspect of the test hypothesis is untrue, the usage advocated by Fisher, a large *P*‐value does not support that it is true, because it says nothing about other test hypotheses (in this case, difference between interventions) with which the data may be compatible. It is, therefore, incorrect to conclude anything substantive from a large *P*‐value. Unfortunately, the incorrect interpretation that a large *P*‐value indicates that an effect or association is absent appears common in clinical trials reporting.^5^

An emphasis on confidence intervals may mitigate some of the limitations of reasoning based on hypothesis tests, even if their exact meaning is unintuitive. This is a popular view,^6^ and is generally encouraged by relevant reporting guidelines. Considering the authors' estimate of the relative incidence risk (which they express as odds ratio) of at least one relapse, which they state as 1.40 (95% CI: 0.40, 4.96, *P* = 0.60), the confidence interval represents, in simple terms, the set of values of the parameter of interest (test hypotheses) with which the data are reasonably consistent; in some sense, those values of the parameter that the data at hand cannot rule out. Although I profess no expertise with the assessment of intervention effect sizes in neurology, at face value the interval is fairly wide; the data are consistent with benefit of the 6‐weekly intervention of about 2.5 reduction (1/0.40) in the odds of relapse, or detriment of about 5 times increase in the odds of relapse. The extent of evidence this study provided in support of the 6‐weekly intervention depends strongly on a contextual interpretation of all the effects within the nominated uncertainty interval, which was not provided by the authors.

Regarding their research objective, the authors state^1^:

Our hypothesis was that the “short” prednisolone course would achieve similar resolution of clinical signs per bout of SRMA and have similar rate of relapse of the disease.

Setting aside that this is a prediction, not a hypothesis, this objective concerns the similarity of interventions, so the authors' interpretation of their hypothesis tests could be considered reversal of a burden of proof. If an investigator were to assume, for the sake of argument, that the effectiveness of a new intervention was identical to an existing one, and then look to some data to overturn this assumption (as implemented by nil hypothesis testing), failing to do so should not be convincing of the similarity of the interventions, because the similarity was assumed in the first place. Under this logic, if an investigator desired to argue that a novel treatment is equivalent to an existing one, they should ideally collect very little data (ie, ensure that their design has low power), which would guarantee a large *P*‐value and assure that their starting assumption would not be overturned; a perverse incentive. Instead, we should expect that effectiveness is presumed to be different, and demand that positive evidence be provided to support that the novel treatment is sufficiently similar to the existing treatment to be practically exchangeable. This is the basic premise of equivalence evaluation, an early subject in modern biostatistics which is extensively explored.^7^ The equivalence framework highlights the importance of substantive interpretation of effect estimates and their uncertainty, including at the design stage.

The conflation of “not statistically significant” with “not different” is an apparently common, but fundamental, error. The apparent effect of this and related misinterpretations of frequentist statistics have led to recent calls for major reform.^8^ For the majority of situations, I contend that veterinary clinical researchers would be wise to set aside “significance,” *P*‐value thresholds, and related concepts altogether, and focus on estimates and their uncertainty.
